# Infinitely Many Weak Solutions of the *p*-Laplacian Equation with Nonlinear Boundary Conditions

**DOI:** 10.1155/2014/194310

**Published:** 2014-01-14

**Authors:** Feng-Yun Lu, Gui-Qian Deng

**Affiliations:** ^1^Xingyi Normal University for Nationalities, Xingyi, Guizhou 562400, China; ^2^Human Resources and Social Security Bureau, Buyi and Miao Autonomous Prefecture in Southwest Guizhou, Guizhou 562400, China

## Abstract

We study the following *p*-Laplacian equation with nonlinear boundary conditions: −Δ_*p*_ *u* + *μ*(*x*) | *u*|^*p*−2^ *u* = *f*(*x*, *u*) + *g*(*x*, *u*), *x* ∈ Ω, | ∇*u*|^*p*−2^∂*u*/∂*n* = *η* | *u*|^*p*−2^ *u* and *x* ∈ ∂Ω, where Ω is a bounded domain in ℝ^*N*^ with smooth boundary ∂Ω. We prove that the equation has infinitely many weak solutions by using the variant fountain theorem due to Zou (2001) and *f*, *g* do not need to satisfy the (*P*.*S*) or (*P*.S*) condition.

## 1. Introduction

In this paper, we study the following  *p*-Laplacian equation:

(1) $$\begin{array}{c} -\Delta_{p}u+\mu \left(x\right){\left|u\right|}^{p-2}u=f\left(x,u\right)+g\left(x,u\right),\;x\in \Omega ,\\ {\left|\nabla u\right|}^{p-2}\frac{\partial u}{\partial n}=\eta {\left|u\right|}^{p-2}u,\;x\in \partial \Omega , \end{array}$$

where  *Ω*  is a bounded domain in  ℝ^*N*^  with smooth boundary  ∂*Ω*  and  ∂/∂*n*  is the outer normal derivative, − Δ_*p*_ *u* = div(|∇*u*|^*p*−2^∇*u*)  is the  *p*-Laplacian with  1 < *p* < *N*,  *η*  is real parameter, and

(2) $$\begin{array}{c} \mu \left(x\right)\in L^{\infty }\left(\Omega \right)\;\;\text{satisfying}\;\;\text{ess}\;\;\underset{x\in \bar{\Omega }}{\inf}\;\mu \left(x\right)>0. \end{array}$$

The perturbation functions  *f*, *g*  satisfy the following conditions:

(F1) $\;f,g\in C(\bar{\Omega }\times \mathbb{R},\mathbb{R})$ are odd in  *u*;
(F2) there exist *σ*, *δ* ∈ (1, *p*), *c*_1_ > 0, *c*_2_ > 0, *c*_3_ > 0 such that
  (3) c1|u|σ≤f(x,u)u≤c2|u|σ+c3|u|δ,for  a.e.  x∈Ω  and  u∈ℝ.
(F3) There exists *p* < *q* < p* (where p* = *pN*/*N* − *p*) such that |*g*(*x*, *u*)|≤*c*(1 + |*u*|^*q*^) for a.e.  *x* ∈ *Ω*  and  *u* ∈ ℝ. Moreover, lim_*u*→0_ *g*(*x*, *u*)/|*u*|^*p*−1^ = 0 uniformly for  *x* ∈ *Ω*.
(F4) Assume that one of the following conditions hold:
  (1) lim_|*u*|→*∞*_ *g*(*x*, *u*)/|*u*|^*p*−2^ *u* = 0 uniformly for  *x* ∈ *Ω*;
  (2) lim_|*u*|→*∞*_ *g*(*x*, *u*)/|*u*|^*p*−2^ *u* = −*∞* uniformly for  *x* ∈ *Ω*; furthermore, *f*(*x*, *u*)/|*u*|^*p*−2^ *u* and *g*(*x*, *u*)/|*u*|^*p*−2^ *u* are decreasing in  *u*  for  *u*  is large enough;
  (3) lim_|*u*|→*∞*_ *g*(*x*, *u*)/|*u*|^*p*−2^ *u* = *∞* uniformly for  *x* ∈ *Ω*; *g*(*x*, *u*)/|*u*|^*p*−2^ *u* is increasing in  *u*  for  *u*  is large enough; moreover, there exists *α* > max{*σ*, *δ*} such that
    (4) $$\begin{array}{c} \underset{\left|u\right|\to \infty }{\mathrm{liminf}}\frac{g\left(x,u\right)u-pG\left(x,u\right)}{{\left|u\right|}^{\alpha }}\geq c>0\;\text{uniformly}\;\;\text{for}\;\;x\in \Omega , \end{array}$$

where  *G*(*x*, *u*) = ∫_0_^*u*^ *g*(*x*, *t*)*dt*.

**Remark 1**

The above conditions were given in Zou [1] for the semilinear case  *p* = 2.

**Remark 2**

A simple example which satisfies (F1)–(F4) is

(5) $$\begin{array}{c} \;f\left(x,u\right)+g\left(x,u\right)=\mu {\left|u\right|}^{r-2}u+\gamma {\left|u\right|}^{s-2}u, \end{array}$$

where  1 < *r* < *p* < *s* < p*.

Equation (1) is posed in the framework of the *Sobolev* space

(6) $$\begin{array}{c} W^{1,p}\left(\Omega \right)=\left\{u\in L^{p}\left(\Omega \right):\int_{\Omega }{\left|\nabla u\right|}^{p}dx<\infty \right\}, \end{array}$$

with the norm

(7) $$\begin{array}{c} ||u||={\left(\int_{\Omega }\left({\left|\nabla u\right|}^{p}+\mu \left(x\right){\left|u\right|}^{p}\right)dx\right)}^{1/p}. \end{array}$$

The corresponding energy functional of (1) is defined by

(8) Φ(u)=1p∫Ω(|∇u|p+μ(x)|u|p)dx−∫ΩF(x,u)dx−∫ΩG(x,u)dx−ηp∫∂Ω|u|pds,

for  *u* ∈ *W* ^1,*p*^(*Ω*), where  *F*(*x*, *u*) = ∫_0_^*u*^ *f*(*x*, *t*)*dt*  and  *ds*  is the measure on the boundary. It is easy to see that Φ ∈ *C* ^1^(*W* ^1,*p*^(*Ω*), ℝ) and

(9) 〈Φ′(u),v〉 =∫Ω(|∇u|p−2∇u∇v+μ(x)|u|p−2uv)dx  −∫Ωf(x,u)vdx−∫Ωg(x,u)vdx−η∫∂Ω|u|p−2uvds,

for all  *u*, *v* ∈ *W* ^1,*p*^(*Ω*). It is well-known that the weak solution of (1) corresponds to the critical point of the energy functional Φ on  *W* ^1,*p*^(*Ω*).

**Remark 3**

Under condition (2), it is easy to check that norm (7) is equivalent to the usual one, that is, the norm defined in (7) with *μ*(*x*) ≡ 1.

In [2], the author researched (1) (*η* = 0) and obtained the existence of infinitely many weak solutions. Moreover, the existence of three solutions for (1) (*η* = 0, *p* > *N*) was researched in [3] by using a three-critical-point theorem due to Ricceri [4]. Also, some authors researched and obtained the existence of infinitely many weak solution without requiring any symmetric conditions and also with discontinuous nonlinearities; see [5, 6]. Recently, this equation was studied by J.-H. Zhao and P.-H. Zhao [7] via Bartsch's dual fountain theorem in [8] and obtained the existence of infinitely many weak solutions for (1) under the case of Remark 2. They obtained the following theorem.

**Theorem A**

Let  *f*(*x*, *t*) + *g*(*x*, *t*) = *μ* | *u*|^*r*−2^ *u* + *γ* | *u*|^*s*−2^ *u*, where  1 < *r* < *p* < *s* < p*. Then there exists a constant  Λ > 0  such that, for any  *η* < Λ,

1. for any  *γ* > , *μ* ∈ ℝ, (1) has a sequence of solutions  *u*_*k*_  such that  Φ(*u*_*k*_) → *∞*  as  *k* → *∞*;
2. for any  *μ* > 0, *γ* ∈ ℝ, (1) has a sequence of solutions  *v*_*k*_  such that  Φ(*v*_*k*_) → 0^−^  as  *k* → *∞*.

The main ingredient for the proof of the above theorem is a dual fountain theorem in [8]. It should be noted that the  (*P*.*S*)  or  (*P*.S*)  condition and its variants play an important role in this theorem and its application. While the variant fountain theorem in Zou [1] does not need not the  (*P*.*S*)  or  (*P*.S*)  condition, we obtain the following generalized result by using Zou's theorem.

**Theorem 4**

Assume that (F1)–(F4) hold; then there exists a constant  Λ > 0  such that, for any  *η* < Λ, (1) has infinitely many weak solutions  {*u*_*k*_}  satisfying

(10) $$\begin{array}{c} \Phi \left(u_{k}\right)⟶0^{-}\;as\;\;k⟶\infty . \end{array}$$

This paper is organized as follows. In Section 2, we recall some preliminary theorems and lemmas. In Section 3, we give the proof of Theorem 4.

## 2. Preliminaries

In what follows, we make use of the following notations:  *E*  (or  *W* ^1,*p*^(*Ω*)) denotes Banach space with the norm  ||·||;  E*  denotes the conjugate space for  *E*;  *L* ^*p*^(*Ω*)  denotes Lebesgue space with the norm |·|_*p*_; 〈·, ·〉 is the dual pairing of the spaces  E* and  *E*; we denote by → (resp., ⇀) the strong (resp., weak) convergence; *c*, *c*_1_, *c*_2_,… denote (possibly different) positive constants.

For completeness, we first recall the variant fountain theorem in Zou [1]. Let  *E*  be a Banach space with norm ||·|| and $E=\bar{{⊕}_{j\in N}X_{j}}$ with dim*X*_*j*_ < *∞* for any  *j* ∈ *N*. Set  *Y*_*k*_ = ⊕_*j*=0_^*k*^ *X*_*j*_, $Z_{k}=\bar{{⊕}_{j=k}^{\infty }X_{j}}$.

**Theorem 5 (see [1, Theorem 2.2])**

The *C* ^1^-functional Φ_*λ*_ : *E* → ℝ defined by Φ_*λ*_(*u*) = *A*(*u*) − *λB*(*u*),  *λ* ∈ [1,2], satisfies

(A1) Φ_*λ*_ maps bounded sets to bounded sets uniformly for  *λ* ∈ [1,2]; furthermore, Φ_*λ*_(−*u*) = Φ_*λ*_(*u*) for all (*λ*, *u*)∈[1,2] × *E*.
(A2) *B*(*u*) ≥ 0 for all  *u* ∈ *E*; *B*(*u*) → *∞* as ||*u*|| → *∞*  on any finite dimensional subspace of  *E*.
(A3) There exists *ρ*_*k*_ > *r*_*k*_ > 0 such that
  (11) $$\begin{array}{c} a_{k}\left(\lambda \right):=\underset{u\in Z_{k},||u||=\rho_{k}}{\inf}\Phi_{\lambda }\left(u\right)\geq 0>b_{k}\left(\lambda \right):=\underset{u\in Y_{k},||u||=r_{k}}{\max}\Phi_{\lambda }\left(u\right); \end{array}$$

for all  *λ* ∈ [1,2],

(12) dk(λ):=infu∈Zk,||u||≤ρkΦλ(u)⟶0 as  k⟶∞  uniformly  for  λ∈[1,2].

Then there exist *λ*_*n*_ → 1, *u*(*λ*_*n*_) ∈ *Y*_*n*_, such that

(13) Φ′λn ∣ Yn(u(λn))=0, Φλn(u(λn))⟶ck∈[dk(2),bk(1)] as  n⟶∞.

Particularly, if {*u*(*λ*_*n*_)} has a convergent subsequence for every  *k*, then  Φ_1_  has infinitely many nontrivial critical points {*u*_*k*_} ∈ *E*∖{0} satisfying Φ_1_(*u*_*k*_) → 0^−^ as *k* → *∞*.

**Remark 6**

Obviously, the sequence  {*u*(*λ*_*n*_)}  is a  (*P*.S*)  sequence.

For our working space *E* = *W* ^1,*p*^(*Ω*), *E* is a reflexive and separable Banach space; then there are  *e*_*j*_ ∈ *E*  and *e*_j_* ∈ E* such that

(14)   E=span{ej:j=1,2,…}¯,  E∗=span{ej∗:j=1,2,…}¯,〈ej∗,ej〉={1,i=j,0,i≠j.

We write  *X*_*j*_ : = span{*e*_*j*_}; then  *Y*_*k*_, *Z*_*k*_  can be defined as that in the beginning of Theorem 5. Consider  Φ_*λ*_ : *E* → ℝ defined by

(15) Φλ(u):=1p||u||p−∫ΩG(x,u)dx−ηp∫∂Ω|u|pds −λ∫ΩF(x,u)dx:=A(u)−λB(u), λ∈[1,2].

Then  *B*(*u*) ≥ 0  for all  *u* ∈ *E*;  *B*(*u*) → *∞*  as  ||*u*|| → *∞*  on any finite dimensional subspace of  *E*;  Φ_*λ*_(−*u*) = Φ_*λ*_(*u*)  for all  *λ* ∈ [1,2], *u* ∈ *E*.  We need the following lemmas.

**Lemma (see [7, Lemma 3.5])**

If  1 ≤ *q* < p*, then one has

(16) $$\begin{array}{c} \beta_{k}:=\underset{u\in Z_{k},||u||=1}{\sup}{\left|u\right|}_{q}⟶0\;as\;\;k⟶\infty . \end{array}$$

## 3. Proof of Theorem 4

First, we check the condition of Theorem 5.

**Lemma 8**

Assume (F1)–(F3); then (A1)–(A3) hold.

**Proof**

(A1) and (A2) are obvious. Let *n* > *k* > 2; we assume that *σ* ≤ *δ*  and define

(17) $$\begin{array}{c} \beta_{k}\left(\sigma \right):=\underset{u\in Z_{k},||u||=1}{\sup}{\left|u\right|}_{\sigma },\;\;\beta_{k}\left(\delta \right):=\underset{u\in Z_{k},||u||=1}{\sup}{\left|u\right|}_{\delta }. \end{array}$$

Observe that

(18) $$\begin{array}{c} {\left|u\right|}_{\sigma }\leq \beta_{k}\left(\sigma \right)||u||,\;\;{\left|u\right|}_{\delta }\leq \beta_{k}\left(\delta \right)||u||, \end{array}$$

for any *u* ∈ *Z*_*k*_. Note that *q* < p*; there exists a constant *c* > 0 such that

(19) $$\begin{array}{c} {\left|u\right|}_{q}\leq c||u||. \end{array}$$

By the* Sobolev *trace imbedding inequality, we have

(20) $$\begin{array}{c} {\left|u\right|}_{L^{p}\left(\partial \Omega \right)}^{p}\leq K{||u||}^{p}. \end{array}$$

Then we take  Λ* = 1/4*K*  such that, for all  *η* < Λ*,

(21) $$\begin{array}{c} \frac{\eta }{p}{\left|u\right|}_{L^{p}\left(\partial \Omega \right)}^{p}\leq \frac{1}{4p}{||u||}^{p}. \end{array}$$

By (F3), for any  *ɛ* > 0, there exists  *c*_*ɛ*_  such that

(22) $$\begin{array}{c} \;\left|G\left(x,u\right)\right|\leq ɛ{\left|u\right|}^{p}+c_{ɛ}{\left|u\right|}^{q}. \end{array}$$

Then, by (F1)–(F3) and (18)–(21), we obtain

(23) Φλ(u)=1p||u||p−∫ΩG(x,u)dx−ηp∫∂Ω|u|pds −λ∫ΩF(x,u)dx≥34p||u||p−ɛ|u|pp−cɛ|u|qq−c|u|σσ−c|u|δδ≥34p||u||p−ɛc||u||p−c||u||q−cβk(σ)σ||u||σ −cβk(δ)δ||u||δ.

Note that *p* < *q*; we may choose *ɛ* > 0 and *R* > 0 sufficiently small that

(24) $$\begin{array}{c} \frac{1}{4p}{||u||}^{p}-ɛc{||u||}^{p}-c{||u||}^{q}\geq 0 \end{array}$$

holds true for any  *u* ∈ *W* ^1,*p*^(*Ω*)  with  ||*u*|| ≤ *R*. So we have

(25) $$\begin{array}{c} \Phi_{\lambda }\left(u\right)\geq \frac{1}{2p}{||u||}^{p}-c\beta_{k}{\left(\sigma \right)}^{\sigma }{||u||}^{\sigma }-c\beta_{k}{\left(\delta \right)}^{\delta }{||u||}^{\delta }, \end{array}$$

for any *u* ∈ *Z*_*k*_  with ||*u*|| ≤ *R*. Choosing

(26) $$\begin{array}{c} \rho_{k}:={\left(4pc\beta_{k}{\left(\sigma \right)}^{\sigma }+4pc\beta_{k}{\left(\delta \right)}^{\delta }\right)}^{1/\left(p-\delta \right)}, \end{array}$$

by Lemma 7, for  *β*_*k*_(*σ*) → 0,  *β*_*k*_(*δ*) → 0  as  *k* → *∞*, it follows that  *ρ*_*k*_ → 0  as  *k* → *∞*, so there exists  *k*_0_  such that *ρ*_*k*_ ≤ *R* when *k* ≥ *k*_0_. Thus, for *k* ≥ *k*_0_, *u* ∈ *Z*_*k*_, and ||*u*|| = *ρ*_*k*_, we have Φ_*λ*_(*u*) ≥ *ρ*_*k*_^*p*^/4*p* > 0; then *a*_*k*_(*λ*) ≥ 0 for all *λ* ∈ [1,2].

On the other hand, if *u* ∈ *Y*_*k*_  with ||*u*|| being small enough, since all the norms are equivalent on the finite dimensional space and  *σ* < *p*, then  *b*_*k*_(*λ*) < 0  for all  *λ* ∈ [1,2].

Furthermore, if *u* ∈ *Z*_*k*_ with ||*u*|| ≤ *ρ*_*k*_, *k* ≥ *k*_0_, we see that

(27) $$\begin{array}{c} \;\Phi_{\lambda }\left(u\right)\geq -c\beta_{k}{\left(\sigma \right)}^{\sigma }\rho_{k}^{\sigma }-c\beta_{k}{\left(\delta \right)}^{\delta }\rho_{k}^{\delta }⟶0\;\text{as}\;\;k⟶\infty . \end{array}$$

Therefore, *d*_*k*_(*λ*) → 0 as *k* → *∞*. Thus, (A3) holds.

By Theorem 5, we have the following lemma.

**Lemma 9**

There exist *λ*_*n*_ → 1 and *u*(*λ*_*n*_) ∈ *Y*_*n*_ such that

(28) Φ′λn|Yn(u(λn))=0,Φλn(u(λn))⟶ck∈[dk(2),bk(1)] as  n⟶∞.

In order to complete our proof of Theorem 4, by a standard argument (see the proof of Lemma 3.4 in Zhao [7]), we only need to show that {*u*(*λ*_*n*_)} is bounded.

**Lemma 10**

  {*u*(*λ*_*n*_)} is bounded in  *W* ^1,*p*^(*Ω*).

**Proof**

Since Φ_*λ*_*n*__′|_*Y*_*n*__(*u*(*λ*_*n*_)) = 0, then

(29) 1−η∫∂Ω|u(λn)|p||u(λn)||pds =∫Ωλnf(x,u(λn)u(λn))+g(x,u(λn))u(λn)||u(λn)||pdx.

We can choose 0 < Λ < Λ* and if *η* < Λ such that 1 − *ηK* > 0. If, up to a subsequence, ||*u*(*λ*_*n*_)|| → *∞* as *n* → *∞*, then, by (F2),

(30) $$\begin{array}{c} 1+\left|\eta \right|K\geq \int_{\Omega }\frac{g\left(x,u\left(\lambda_{n}\right)\right)u\left(\lambda_{n}\right)}{{||u\left(\lambda_{n}\right)||}^{p}}dx\geq \frac{1}{2}\left(1-\eta K\right), \end{array}$$

for *n* is large enough. Obviously, it is a condition if (F4)(1) holds.

Otherwise, we set *w*_*n*_ = *u*(*λ*_*n*_)/||*u*(*λ*_*n*_)||; then, up to a subsequence,

(31) $$\begin{array}{c} w_{n}⇀w\;\;\text{in}\;\;E,\\ w_{n}⟶w\;\;\text{in}\;\;L^{t}\left(\Omega \right)\;\text{for}\;\;1\leq t<p^{*},\\ w_{n}\left(x\right)⟶w\left(x\right)\;\text{a}.\text{e}.\;\;x\in \Omega . \end{array}$$

If *w* ≠ 0 in *E* and lim_|*u*|→*∞*_ *g*(*x*, *u*)/|*u*|^*p*−2^ *u* = −*∞* in (F4)(2), then, for *n* is large enough, by Fatou's Lemma, we have that

(32) −12(1−ηK) ≥∫Ω−g(x,u(λn))u(λn)|u(λn)|p|wn|pdx ≥c+∫{x∈Ω:w≠0,|u(λn)|≥c}−g(x,u(λn))u(λn)|u(λn)|p|wn|pdx ⟶∞;

this is a contradiction. It is similar if lim_|*u*|→*∞*_ *g*(*x*, *u*)/|*u*|^*p*−2^ *u* = *∞* in (F4)(3). Thus, *w* = 0.

Let *t*_*n*_ ∈ [0,1] such that

(33) $$\begin{array}{c} \Phi_{\lambda_{n}}\left(t_{n}u\left(\lambda_{n}\right)\right):=\underset{t\in \left[0,1\right]}{\max}\Phi_{\lambda_{n}}\left(tu\left(\lambda_{n}\right)\right). \end{array}$$

For any  *c* > 0  large enough, and ${\bar{w}}_{n}:={\left(2pc\right)}^{1/p}w_{n}$, for *n* is large enough, we have that

(34) Φλn(tnu(λn))≥Φλn(w¯n)=2c−∫ΩG(x,w¯n)dx−ηp∫∂Ω|w¯n|pds −λn∫ΩF(x,w¯n)dx≥c,

which implies that lim_*n*→*∞*_Φ_*λ*_*n*__(*t*_*n*_ *u*(*λ*_*n*_)) → *∞*. Obviously,

(35) $$\begin{array}{c} \langle \Phi_{\lambda_{n}}^{\prime }\left(t_{n}u\left(\lambda_{n}\right)\right),t_{n}u\left(\lambda_{n}\right)\rangle =0. \end{array}$$

It follows that

(36) ∞=limn→∞(Φλn(tnu(λn))−1p〈Φ′λn(tnu(λn)),tnu(λn)〉)≤limn→∞λn∫Ω(1pf(x,tnu(λn))tnu(λn)         −F(x,tnu(λn)))dx +∫Ω(1pg(x,tnu(λn))tnu(λn)−G(x,tnu(λn)))dx.

If (F4)(2) holds, we have that (1/*p*)*f*(*x*, *u*)*u* − *F*(*x*, *u*) and (1/*p*)*g*(*x*, *u*)*u* − *G*(*x*, *u*) are decreasing in  *u*  for *u* is large enough. Therefore,

(37) $$\begin{array}{c} \;\frac{1}{p}f\left(x,su\right)su-F\left(x,su\right)+\frac{1}{p}g\left(x,su\right)su-G\left(x,su\right)\leq c \end{array}$$

for all *s* > 0 and *u* ∈ ℝ; it is a contradiction.

If (F4)(3) holds, then we have that

(38) ∞≤c∫Ω|u(λn)|σdx+∫Ω(1pg(x,u(λn))u(λn)−G(x,u(λn)))dx,

which implies

(39) $$\begin{array}{c} \int_{\Omega }\left(\frac{1}{p}g\left(x,u\left(\lambda_{n}\right)\right)u\left(\lambda_{n}\right)-G\left(x,u\left(\lambda_{n}\right)\right)\right)dx⟶\infty . \end{array}$$

On the other hand, by the property of *u*(*λ*_*n*_), for *n* is large enough, since *α* > max{*δ*, *σ*}, we have that

(40) bk(1) ≥λn∫Ω(1pf(x,u(λn))u(λn)−F(x,u(λn))  )dx  +∫Ω(1pg(x,u(λn))u(λn)−G(x,u(λn)))dx ≥12∫Ω(1pg(x,u(λn))u(λn)−G(x,u(λn)))dx  +12c∫Ω|u(λn)|αdx−12c∫Ω|u(λn)|δdx  −12c∫Ω|u(λn)|σdx ≥c∫Ω(1pg(x,u(λn))u(λn)−G(x,u(λn)))dx−c;

this implies that ∫_*Ω*_((1/*p*)*g*(*x*, *u*(*λ*_*n*_))*u*(*λ*_*n*_) − *G*(*x*, *u*(*λ*_*n*_)))*dx* is bounded, which contradicts (39).

By the above arguments, we have that {*u*(*λ*_*n*_)} is bounded.

**Remark 11**

In fact, our result still holds if we consider a weaker condition than (F4)(2); that is, there is *c* > 0 such that

(41) $$\begin{array}{c} H\left(x,t\right)\leq H\left(x,s\right)+c,\;\;\bar{H}\left(x,t\right)\leq \bar{H}\left(x,s\right)+c \end{array}$$

for all 0 < *s* < *t* or *t* < *s* < 0,  ∀ *x* ∈ *Ω*, where *H*(*x*, *t*) = (1/*p*)*f*(*x*, *t*)*t* − *F*(*x*, *t*) and $\bar{H}(x,t)=\left(1/p\right)g(x,t)t-G(x,t)$.
